# Adaptor protein-2 sigma subunit mutations causing familial hypocalciuric hypercalcaemia type 3 (FHH3) demonstrate genotype–phenotype correlations, codon bias and dominant-negative effects

**DOI:** 10.1093/hmg/ddv226

**Published:** 2015-06-16

**Authors:** Fadil M. Hannan, Sarah A. Howles, Angela Rogers, Treena Cranston, Caroline M. Gorvin, Valerie N. Babinsky, Anita A. Reed, Clare E. Thakker, Detlef Bockenhauer, Rosalind S. Brown, John M. Connell, Jacqueline Cook, Ken Darzy, Sarah Ehtisham, Una Graham, Tony Hulse, Steven J. Hunter, Louise Izatt, Dhavendra Kumar, Malachi J. McKenna, John A. McKnight, Patrick J. Morrison, M. Zulf Mughal, Domhnall O'Halloran, Simon H. Pearce, Mary E. Porteous, Mushtaqur Rahman, Tristan Richardson, Robert Robinson, Isabelle Scheers, Haroon Siddique, William G. van't Hoff, Timothy Wang, Michael P. Whyte, M. Andrew Nesbit, Rajesh V. Thakker

**Affiliations:** 1 Academic Endocrine Unit, Radcliffe Department of Medicine, University of Oxford, Oxford, UK,; 2 Oxford Molecular Genetics Laboratory, Churchill Hospital, Oxford, UK,; 3 Renal Unit, Great Ormond Street Hospital for Children NHS Foundation Trust and UCL Institute of Child Health, London, UK,; 4 Division of Endocrinology, Boston Children's Hospital, Boston, MA, USA,; 5 School of Medicine, Ninewells Hospital, University of Dundee, Dundee, UK,; 6 Clinical Genetics Department, Sheffield Children's Hospital NHS Foundation Trust, Sheffield, UK,; 7 Queen Elizabeth II Hospital, Welwyn Garden City, UK,; 8 Department of Paediatric Endocrinology, Royal Manchester Children's Hospital, Manchester, UK,; 9 Regional Centre for Endocrinology and Diabetes, Royal Victoria Hospital, Belfast, UK,; 10 Department of Paediatrics, Evelina London Children's Hospital, St. Thomas’ Hospital, London, UK,; 11 Department of Clinical Genetics, Guy's Hospital, London, UK,; 12 Institute of Cancer and Genetics, University Hospital of Wales, Cardiff, UK,; 13 Department of Endocrinology, St. Vincent's University Hospital, Dublin, Ireland,; 14 Metabolic Unit, Western General Hospital, NHS Lothian and University of Edinburgh, Edinburgh, UK,; 15 Centre for Cancer Research and Cell Biology, Queens University of Belfast, Belfast, UK,; 16 Department of Genetic Medicine, Belfast HSC Trust, Belfast, UK,; 17 Department of Endocrinology, Cork University Hospital, Cork, Ireland,; 18 Institute of Genetic Medicine, Newcastle University, Newcastle upon Tyne, UK,; 19 SE Scotland Genetic Service, Western General Hospital, Edinburgh, UK,; 20 Department of Endocrinology, Northwick Park Hospital, London, UK,; 21 Diabetes and Endocrine Centre, Royal Bournemouth Hospital, Bournemouth, UK,; 22 Department of Endocrinology, Chesterfield Royal Hospital NHS Foundation Trust, Derbyshire, UK,; 23 Pediatric Gastroenterology, Hepatology and Nutrition Unit, Cliniques Universitaires Saint-Luc, Brussels, Belgium,; 24 Department of Endocrinology, Russells Hall Hospital, Dudley, UK,; 25 Department of Clinical Biochemistry, Frimley Park Hospital, Surrey, UK and; 26 Center for Metabolic Bone Disease and Molecular Research, Shriners Hospital for Children, St. Louis, Missouri, USA

## Abstract

The adaptor protein-2 sigma subunit (AP2σ2) is pivotal for clathrin-mediated endocytosis of plasma membrane constituents such as the calcium-sensing receptor (CaSR). Mutations of the AP2σ2 Arg15 residue result in familial hypocalciuric hypercalcaemia type 3 (FHH3), a disorder of extracellular calcium (Ca^2+^_o_) homeostasis. To elucidate the role of AP2σ2 in Ca^2+^_o_ regulation, we investigated 65 FHH probands, without other FHH-associated mutations, for AP2σ2 mutations, characterized their functional consequences and investigated the genetic mechanisms leading to FHH3. AP2σ2 mutations were identified in 17 probands, comprising 5 Arg15Cys, 4 Arg15His and 8 Arg15Leu mutations. A genotype–phenotype correlation was observed with the Arg15Leu mutation leading to marked hypercalcaemia. FHH3 probands harboured additional phenotypes such as cognitive dysfunction. All three FHH3-causing AP2σ2 mutations impaired CaSR signal transduction in a dominant-negative manner. Mutational bias was observed at the AP2σ2 Arg15 residue as other predicted missense substitutions (Arg15Gly, Arg15Pro and Arg15Ser), which also caused CaSR loss-of-function, were not detected in FHH probands, and these mutations were found to reduce the numbers of CaSR-expressing cells. FHH3 probands had significantly greater serum calcium (sCa) and magnesium (sMg) concentrations with reduced urinary calcium to creatinine clearance ratios (CCCR) in comparison with FHH1 probands with CaSR mutations, and a calculated index of sCa × sMg/100 × CCCR, which was ≥ 5.0, had a diagnostic sensitivity and specificity of 83 and 86%, respectively, for FHH3. Thus, our studies demonstrate AP2σ2 mutations to result in a more severe FHH phenotype with genotype–phenotype correlations, and a dominant-negative mechanism of action with mutational bias at the Arg15 residue.

## Introduction

Familial hypocalciuric hypercalcaemia (FHH) is an autosomal dominant disorder of extracellular calcium (Ca^2+^_o_) homeostasis characterized by lifelong mild-to-moderate elevations of serum calcium concentrations, mild hypermagnesaemia, normal or elevated circulating parathyroid hormone (PTH) concentrations and inappropriately low urinary calcium excretion [mean urinary calcium to creatinine clearance ratio (CCCR) <0.01] (1–4). FHH is a genetically heterogeneous disorder comprising three reported variants. FHH types 1 and 2 (FHH1, OMIM #145980; FHH2, OMIM #145981) are due to heterozygous loss-of-function mutations of the calcium-sensing receptor (CaSR) and G-protein, Gα_11_, encoded by the *CASR* and *GNA11* genes, respectively (5–9). CaSR and Gα_11_ are widely expressed, including in the parathyroid glands and kidneys, and play a pivotal role in Ca^2+^_o_ homeostasis by detecting alterations in Ca^2+^_o_ concentrations and initiating multiple intracellular signalling cascades that include phospholipase C-mediated accumulation of inositol 1,4,5-trisphosphate and increases in intracellular calcium (Ca^2+^_i_) concentrations (10), which in turn lead to alterations in PTH secretion and urinary calcium excretion. FHH type 3 (FHH3, OMIM #600740) is associated with heterozygous loss-of-function mutations of *AP2S1*, located on chromosome 19q13.3 (11–14).

*AP2S1* encodes the σ2-subunit of the ubiquitously expressed heterotetrameric adaptor protein-2 (AP2) complex, which also comprises α-, β2- and μ2-subunits. The AP2 complex is a central component of clathrin-coated vesicles and facilitates the endocytosis of plasma membrane constituents such as G-protein-coupled receptors (GPCRs) (15–17). *AP2S1* mutations have been reported in 19 FHH patients and families to date, and these all comprise heterozygous missense substitutions of the AP2 σ2-subunit (AP2σ2) Arg15 residue (Arg15Cys, Arg15His and Arg15Leu) (11,12,14). This Arg residue is located in a positively charged region of AP2σ2 that binds to specific peptide motifs on membrane cargo proteins (18). It is predicted that FHH3-causing Arg15 mutations disrupt binding of the AP2 complex to the intracellular carboxyl terminus of the CaSR, thereby impairing endocytosis of this GPCR (11). This hypothesis is supported by *in vitro* expression studies that have demonstrated *AP2S1* mutations to affect CaSR cell-surface expression and signal transduction (11). These studies of *AP2S1* mutations have highlighted a role for the AP2 endocytic complex in Ca^2+^_o_ homeostasis. To date, 19 FHH3 patients have been reported with AP2σ2 mutations, comprising 8 Arg15Cys, 4 Arg15His and 7 Arg15Leu. To further elucidate the role and spectrum of AP2σ2 mutations in the aetiology of the phenotypic features of FHH3, we conducted studies to characterise the structural/functional consequences of AP2σ2 mutations together with their underlying genetic mechanisms in additional FHH patients, who did not have *CASR* or *GNA11* mutations. Our study identified AP2σ2 mutations in 17 hypercalcaemic probands and their families, and further analysis of these AP2σ2 mutants has revealed the presence of a genotype–phenotype correlation, a mutational bias of the AP2σ2 Arg15 residue with a likely dominant-negative action and a clinical approach to differentiate patients with FHH3 from those with FHH1.

## Results

### AP2S1 mutations and clinical phenotypes

**Figure 1.**
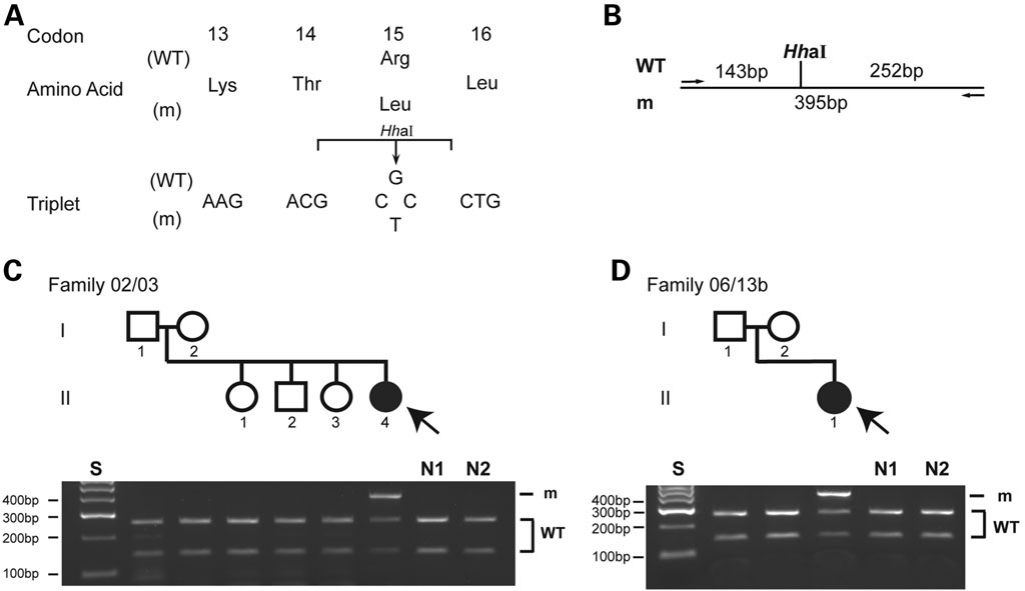
Detection of *de novo AP2S1* mutations in families 02/03 and 06/13b. (**A**) DNA sequence analyses of the probands (arrowed) revealed a G-to-T transversion at codon 15, predicted to result in a missense amino acid substitution of Arg to Leu, and loss of a *Hh*aI restriction endonuclease site. (**B**) Restriction map showing that *Hh*aI digestion would result in two products of 143 and 252 bp from the wild-type (WT) sequence, but would not affect the mutant (m) sequence. PCR and *Hh*aI digestion revealed the probands [individual II.4 of family 02/03 (**C**) and individual II.1 of family 06/13b (**D**)] to be heterozygous for the Arg15Leu mutation. The absence of the Arg15Leu mutation in the unaffected parents of both probands is consistent with the mutation arising *de novo*.

DNA sequence analysis of the entire *AP2S1* 429-bp coding region and 8 exon–intron boundaries was undertaken in 65 unrelated FHH probands without *CASR* or *GNA11* mutations (22 males and 43 females). This revealed the presence of *AP2S1* mutations in 17 probands (7 males and 10 females), thereby representing a >25% *AP2S1* mutation detection rate in this cohort of FHH patients without *CASR* and *GNA11* mutations. The FHH3-associated mutations only affected the AP2σ2 Arg15 (R15) residue and consisted of five Arg15Cys (R15C), four Arg15His (R15H) and eight Arg15Leu (R15L) mutations (Table 1), all of which had previously been reported to represent pathogenic mutations (11). Two unrelated FHH3 female probands, aged 7 and 15 years, harboured Arg15Leu mutations that were demonstrated to be absent in both of their parents, and hence likely to be arising *de novo* (Fig. 1). Four FHH3 subjects had symptoms attributable to hypercalcaemia, including lethargy, constipation, widespread musculoskeletal pain and polydipsia (Table 1). Eleven FHH3 probands had clinical features in addition to hypercalcaemia and hypocalciuria (Table 1). In particular, bone mineral density (BMD) was noted to be low (T-score < −1.0 or Z-score < −2.0) at the lumbar spine or femoral neck in 5 of 10 patients aged 14 to 64 years (Table 1). The low BMD in all five patients was not associated with renal dysfunction, hyperparathyroidism, vitamin D deficiency or thyrotoxicosis. Furthermore, seven FHH3 patients (aged 3–37 years) were noted to have learning disabilities characterized by cognitive deficits and/or behavioural disturbances (Table 1). In addition, the father of a proband with learning disabilities (06/13a, Table 1) also had a cognitive deficit in association with hypercalcaemia. Two of the Arg15Leu probands with cognitive deficits (02/03 and 02/11, Table 1) had short stature with height at or below the third centile, and one of these probands (02/03, Table 1) was found to harbour an atrial septal defect, whereas the other individual (02/11, Table 1) suffered from recurrent episodes of pancreatitis. The pancreatitis in this patient was not due to gallstones or alcohol abuse, and analysis of genes known to be associated with pancreatitis, such as *SPINK1*, *CFTR*, *PRSS1* and *CTRC* (19), did not reveal any mutations.

**Table 1.** Clinical and biochemical findings in 17 FHH probands with *AP2S1* mutations

						Serum					Urine	BMD	
Mutation	Patient	Sex	Family history	Age at presentation/diagnosis (years)	Associated clinical features	Ca^a,b^	Pi^c^	Mg^d^	ALP^e,f^	PTH^g,h^	CCCR^i,j^	LS	FN
Arg15Cys													
	19/12a	M	Yes	48	Nil	2.94^a^	–	0.82	110^e^	45.0^g^	0.009^i^	−1.0^k^	−2.9^k^
	03/13a	M	Yes	22	H	2.96^a^	0.82	–	98^e^	41.0^g^	0.004^i^	–	–
	16/13	M	Yes	37	H, L	2.80^a^	0.76	1.02	68^e^	50.0^g^	0.003^i^	−1.6^k^	−0.2^k^
	07/14	F	Yes	16	B	1.50^b^	1.22	0.95	39^e^	44.0^g^	0.004^i^	−1.2^k^	−1.4^k^
	11/14	M	–	3	L	2.90^a^	1.09	0.91	368^f^	60.0^g^	0.004^i^	–	–
Arg15His													
	08/11	F	Yes	33	H	2.76^a^	0.85	0.99	74^e^	7.4^h^	0.010^i^	−2.1^k^	−1.7^k^
	03/12	F	Yes	44	Nil	2.80^a^	0.86	–	65^e^	5.2^h^	0.009^i^	0.0^k^	−0.4^k^
	19/12b	F	–	19	–	2.74^a^	0.65	–	104^e^	4.6^h^	0.005^i^	–	–
	03/13b	M	Yes	64	Nil	2.72^a^	–	–	103^e^	6.0^h^	–	−0.6^l^	−1.1^l,m^
Arg15Leu													
	02/03	F	No	15	A, L, S	2.90^a^	0.86	1.03	–	4.7^h^	0.015^i^	−4.6^k^	–
	13/08	F	No	<1^o^	L	3.01^a^	0.99	–	242^f^	15.0^g^	–	–	–
	02/11	F	–	14	L, P, S	3.20^a^	1.2	1.02	88^e^	31.0^g^	0.005^i^	−3.0^k^	−2.5^k^
	04/11	M	–	11	L	3.00^a^	0.60	0.89	314^f^	50^g^	0.004^i^	−1.4^k^	−1.4^k^
	09/12	F	–	9	–	2.81^a^	1.0	0.97	298^f^	96^g^	0.002^i^		
	06/13a	M	Yes	9	L	3.10^a^	0.78	–	249^f^	38^g^	0.27^j^	–	–
	06/13b	F	No	7	Nil	3.03^a^	0.87	0.95	285^f^	40.5^g^	0.001^i^	−1.3^k,n^	N
	02/14	F	No	26	H	2.95^a^	1.17	0.98	–	4.0^h^	0.008^i^	–	–

Normal serum ranges (6): albumin adjusted-calcium, ^a^2.10–2.60 mmol/l; ionized calcium, ^b^1.19–1.35 mmol/l; phosphate (Pi), ^c^0.70–1.40 mmol/l; magnesium (Mg), ^d^0.70–1.0 mmol/l; total alkaline phosphatase (ALP) activity, ^e^30–130 U/l, ^f^70–330 U/l; PTH, ^g^10–65 ng/l; ^h^1.3–7.6 pmol/l. Normal urine ranges; calcium-to-creatinine clearance ratio (CCCR), ^i^>0.02; calcium-to-creatinine ratio, ^j^0.3–0.7. ^k^BMD Z-scores are provided for subjects of <50 years old and ^l^BMD T-scores are provided for subjects of >50 years old; ^m^forearm BMD T-score = −2.5 for proband 03/13b; ^n^whole-body BMD Z-score = −2.0 for proband 06/13b; ^o^diagnosed in early infancy. A, atrial septal defect; B, irritable bowel syndrome co-segregating with hypercalcaemia in family 07/14; FN, femoral neck; H, hypercalcaemic symptoms; L, mild-to-moderate learning disability; LS, lumbar spine; N, normal; S, short stature (height <third centile); P, pancreatitis; —, not known.

### Genotype–phenotype correlations at the AP2σ2 Arg15 residue

**Figure 2.**
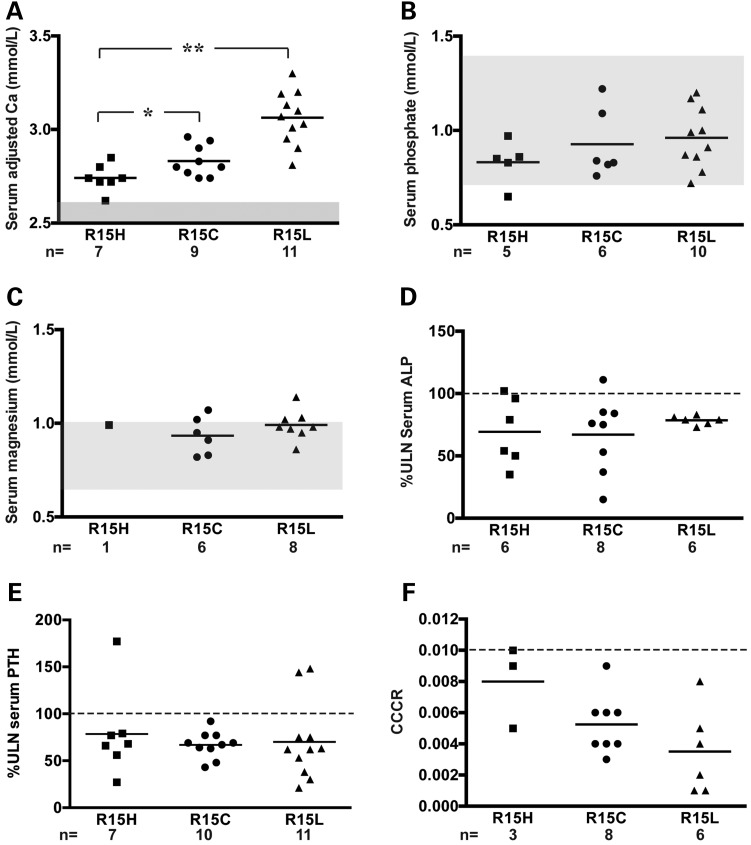
Assessment of genotype–phenotype correlations between probands harbouring Arg15His (R15H), Arg15Cys (R15C) or Arg15Leu (R15L) *AP2S1* mutations. Serum and urine biochemical values are shown as scatter plots. (**A**) Probands with R15L mutations had significantly greater elevations of serum adjusted-calcium concentrations than probands with R15C or R15H mutations. Probands with R15C mutations were significantly more hypercalcaemic than probands with R15H mutations. (**B–F**) No significant differences in serum concentrations of phosphate, magnesium, ALP activity, PTH or urinary CCCR were observed between probands harbouring each of the three *AP2S1* R15 mutations. Mean values for the respective groups are indicated by horizontal solid lines. The normal ranges [mean ± 2 standard deviations (SDs)] for serum calcium, phosphate and magnesium are indicated by the grey areas. The upper limit of normal (ULN) for the assay that was used for serum ALP activity and PTH concentrations are represented by the broken line. For CCCR, the lower limit (<0.01) for the consideration of hypocalciuria is represented by the broken line. *P < 0.05, **P < 0.01.

We examined for phenotypic differences between patients with Arg15Cys, Arg15His or Arg15Leu *AP2S1* mutations. Analysis of biochemical and clinical data available for a total of 27 FHH3 probands [17 from this study and 10 from our previously reported study (11)] revealed that patients with the Arg15Leu mutation had significantly greater elevations of serum albumin adjusted-calcium concentrations when compared with those with the Arg15Cys and Arg15His AP2σ2 mutations (3.06 ± 0.04 mmol/l for Arg15Leu versus 2.83 ± 0.03 mmol/l for Arg15Cys and 2.74 ± 0.03 mmol/l for Arg15His, *P* < 0.01). Moreover, patients with the Arg15Cys mutation had a significantly greater hypercalcaemia (*P* < 0.05) than patients harbouring the Arg15His mutation. Such differences were not observed for other biochemical indices of mineral metabolism (Fig. 2).

### Phenotypic differences between FHH3 and FHH1

**Figure 3.**
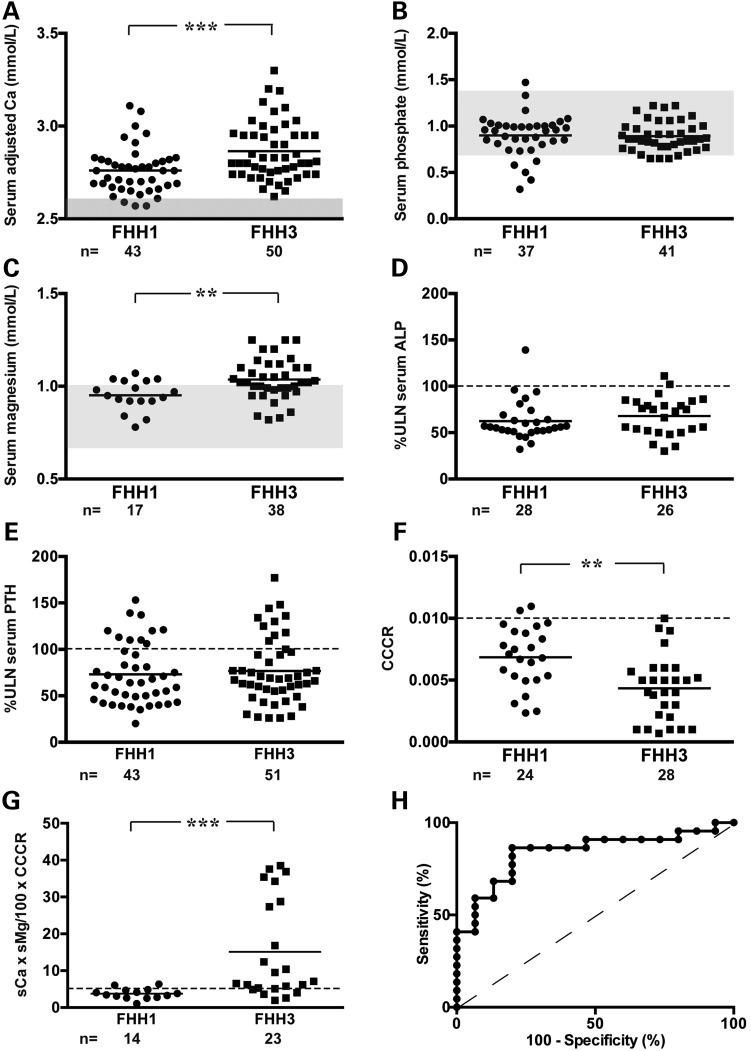
Comparison of biochemical phenotypes between FHH1 and FHH3. Scatter plots of serum concentrations of (**A**) adjusted-calcium, (**B**) phosphate, (**C**) magnesium, (**D**) ALP activity, (**E**) PTH, (**F**) urinary CCCR and (**G**) CMCR index, which is calculated as sCa × sMg/100 × CCCR, for FHH3 probands are shown. Such biochemical values are also provided for an age- and gender-matched cohort of previously reported FHH1 probands with *CASR* mutations (8). All biochemical parameters comprising the CMCR index were measured in millimole per litre. FHH3 probands, when compared with FHH1 probands, had significantly greater elevations of serum adjusted-calcium and magnesium concentrations, reduced CCCR values and elevated CMCR. Mean values for the respective groups are indicated by horizontal solid lines. The normal ranges (mean ± 2 SDs) for serum calcium, phosphate and magnesium are indicated by the grey areas. The ULN for the assay that was used for serum ALP activity and PTH concentrations are represented by the broken line. For CCCR, the lower limit (<0.01) for the consideration of hypocalciuria is represented by the broken line. For CMCR index, the cut-off value of 5.0, above which a diagnosis of FHH3 should be considered, is represented by the broken line. (**H**) ROC of discriminatory power of CMCR index to distinguish between FHH3 and FHH1. The CMCR had an AUC of 0.85, which was significantly greater than that of the reference line (*P* < 0.001). **P < 0.01, ***P < 0.001.

To determine whether FHH3 is associated with any differences in the biochemical phenotype when compared with FHH1, we analysed the serum and urine biochemistry from 51 FHH3 subjects that included affected members of the previously reported multi-generational FHH3 kindreds from Oklahoma (FHH_OK_) and Northern Ireland (FHH_NI_) (20–23), and 43 previously reported FHH1 probands, who all harboured *CASR* mutations (8). This revealed FHH3 patients to have a greater degree of hypercalcaemia than the FHH1 patients (serum adjusted-calcium = 2.87 ± 0.02 mmol/l for FHH3 versus 2.76 ± 0.02 mmol/l for FHH1, *P* < 0.001) (Fig. 3). FHH3 patients when compared with FHH1 patients also had significantly marked hypermagnesaemia (serum magnesium = 1.04 ± 0.02 mmol/l for FHH3 versus 0.95 ± 0.02 mmol/l for FHH1, *P* < 0.01) and hypocalciuria (CCCR = 0.004 ± 0.001 for FHH3 versus 0.007 ± 0.001 for FHH1, *P* < 0.01) (Fig. 3). There were no significant differences in the serum concentrations of phosphate, alkaline phosphatase (ALP) activity or PTH between the FHH3 and FHH1 patients (Fig. 3). As serum adjusted-calcium, serum magnesium and CCCR values were significantly different between FHH3 and FHH1 patients, we investigated whether the combined use of these biochemical parameters could be utilized to discriminate between these two hypercalcaemic disorders. Based on the observation that serum adjusted-calcium (sCa) and serum magnesium (sMg) values are elevated, whereas CCCR is reduced in FHH3, a calculated index (sCa × sMg/100 × CCCR), designated CMCR, was determined for each of the subjects in the FHH1 and FHH3 groups (Fig. 3) with all biochemical parameters being measured in millimole per litre. Receiver–operator curve (ROC) analysis of the CMCR index (Fig. 3) revealed an area-under-the curve (AUC) of 0.85 (*P* < 0.001) and an optimal cut-off value of 5.0 for discriminating between FHH3 and FHH1, which provides a diagnostic sensitivity of 83% [95% confidence interval (CI) = 61–95] and specificity of 86% (95% CI = 57–98), and positive and negative predictive values of 90% (95% CI = 70–98) and 80% (95% CI = 52–95), respectively.

### Analysis of AP2σ2 codon 15 mutation bias

**Figure 4.**
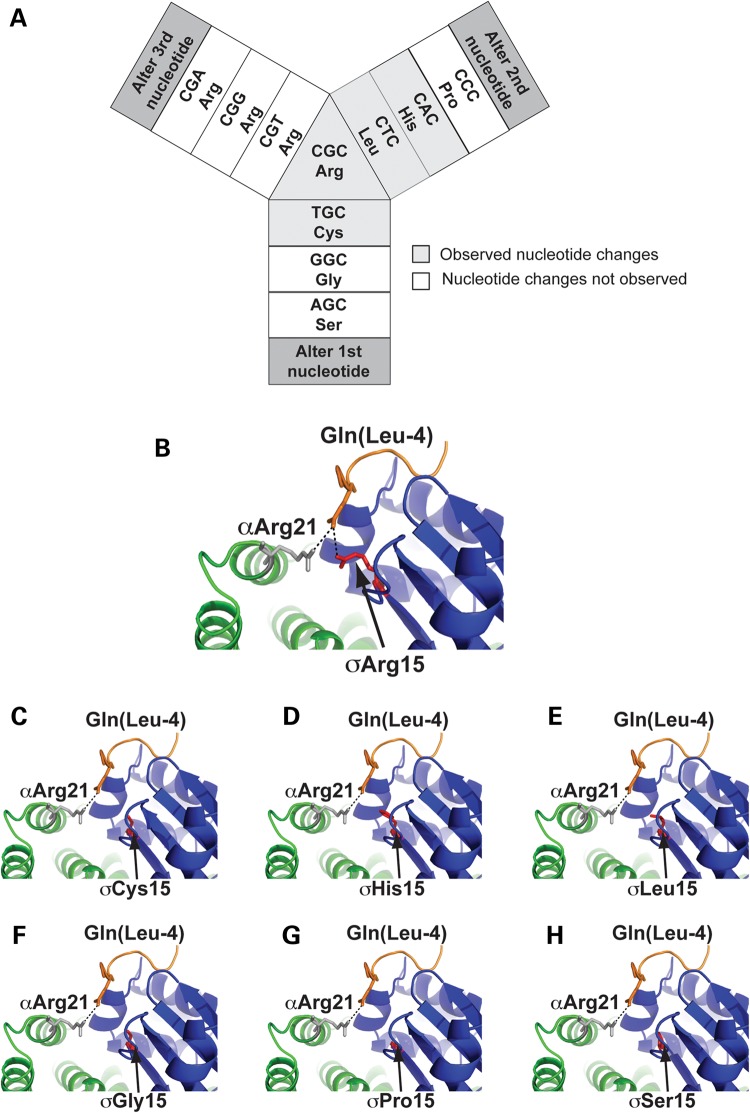
Observed and predicted mutations at the *AP2S1* Arg15 (R15) residue. **(A)** Schematic representation of possible nucleotide and amino acid substitutions affecting codon 15. Substitutions affecting the first or second nucleotide of codon 15 (CGC) are predicted to lead to one of six possible non-synonymous mutations or variants, which are Cys15 (TGC), Gly15 (GGC), His15 (CAC), Leu15 (CTC), Pro15 (CCC) or Ser15 (AGC). Substitutions affecting the third nucleotide of the CGC triplet are predicted to lead to synonymous variants only. **(B)** Crystal structure of the α-subunit (green) and σ2-subunit (blue) of the AP2 heterotetrameric complex bound to an acidic (Gln-containing) dileucine cargo protein motif [PDB file 2JKR (18)]. The key polar contacts (black dashed lines) between the σ2-subunit Arg15 residue (σArg15, red) and α-subunit Arg21 residue (αArg21, grey), and a Gln residue located four residues from the first Leu residue of the acidic dileucine motif [Gln(Leu-4), orange], are shown. **(C–G)** Structural analysis of six potential Arg15 mutations demonstrating that the observed mutants (Cys15, His15 and Leu15) and non-observed mutants (Gly15, Pro15 and Ser15) are all predicted to result in the loss of the key polar contact with the cargo protein Gln (Leu-4) residue.

The present study has identified 17 hypercalcaemic patients with *AP2S1* mutations that all affected the AP2σ2 Arg15 residue, and previous studies have reported 19 *AP2S1* mutations that all affect the Arg15 residue (11,12,14) yielding a total of 36 FHH3-causing mutations identified to date. These 36 *AP2S1* mutations involving substitutions of Arg15 comprise 13 Arg15Cys, 8 Arg15His and 15 Arg15Leu. Analysis of the DNA sequence of the Arg15 codon reveals it to be CGC indicating that missense substitutions affecting the first or second nucleotides would be predicted to be non-synonymous and lead to one of six possible amino acid substitutions, which comprise Cys, Gly, His, Leu, Pro and Ser (Fig. 4). This expected observation of six different amino acid substitutions contrasts significantly (*P* < 0.0001, Chi-squared test) with the three Arg15-mutant substitutions observed in FHH3 patients.

**Figure 5.**
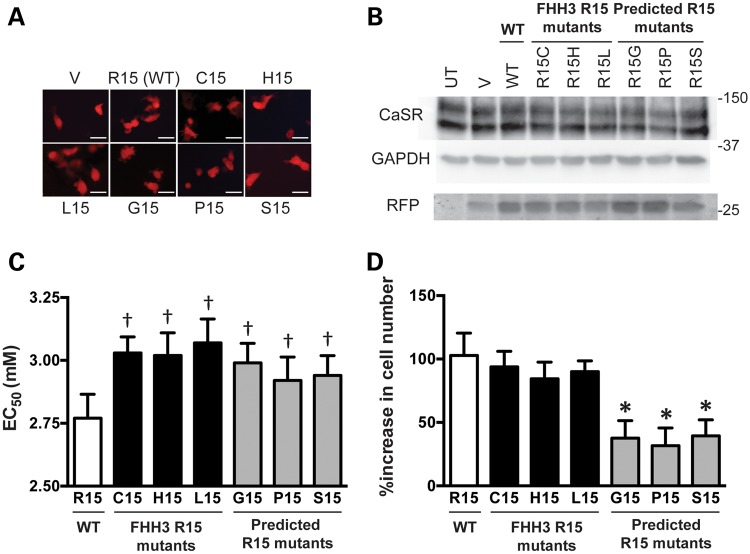
Functional expression of *AP2S1* Arg15 (R15) mutants. (**A**) Fluorescence microscopy of HEK293 cells stably transfected with CaSR and transiently transfected with wild-type R15 (WT-R15), and FHH3-associated mutants (Cys15, C15; His15, H15; Leu15, L15) or other possible R15 mutants (Fig. 4) (Gly15, G15; Pro15, P15; Ser15, S15), or pBI-CMV4-RFP expression vector only (V). RFP expression in these cells indicates successful transfection and expression by these constructs. Bar indicates 20 μm. (**B**) Western blot analysis of whole-cell lysates using anti-CaSR, anti-GAPDH and anti-RFP antibodies. The FHH3-mutant AP2σ2 and predicted possible R15 mutant proteins were expressed at similar levels. UT, untransfected cells. (**C**) Measurement of Ca^2+^_i_ responses following stimulation with varying Ca^2+^_o_ concentrations revealed cells expressing observed FHH3-associated mutants or the non-observed possible R15 mutants (Fig. 4) to have significantly raised EC_50_ values when compared with cells expressing the wild-type AP2σ2 (WT-R15) protein. Results are from eight to ten assays and three independent transfections. (**D**) Growth of cells expressing WT or mutant AP2σ2 proteins. Cells expressing the non-observed possible R15 mutants showed a significantly reduced percentage increase in cell numbers over a 24-h period when compared with cells expressing WT or FHH3-associated mutant AP2σ2 proteins, consistent with an impairment of proliferation. Results are from 11 to 18 assays and 4 independent transfections. *P < 0.05 and ^†^*P* < 0.0001.

To investigate the potential occurrence of the Arg15Cys, Arg15His and Arg15Leu, and the absence of Arg15Gly, Arg15Pro and Arg15Ser variants, we assessed the effects of all six potential Arg15 mutants on the structure and function of AP2. Analysis of the AP2 complex crystal structure predicted that all the six Arg15 missense substitutions, including the Arg15Gly, Arg15Pro and Arg15Ser variants that have not been observed in FHH3, would impair AP2 complex function by disrupting a key polar contact between the AP2 complex and the cargo protein dileucine recognition motif (Fig. 4). Thus, all the six Arg15 missense substitutions would significantly alter the structure, and we therefore determined their functional consequences on CaSR activity, by expressing them in HEK293 cells that stably expressed the CaSR (HEK-CaSR) (9,11). Transient transfection of the wild-type or mutant *AP2S1*-pBI-CMV4-RFP expression constructs, or vector containing the red fluorescence protein (RFP) reporter gene alone, was undertaken in the HEK-CaSR cells (9,11). Expression of the CaSR and RFP, which represents a surrogate of AP2σ2 expression, was detected by immunofluorescence and western blotting of whole-cell lysates (Fig. 5), and the responses of Ca^2+^_i_ concentrations to alterations in Ca^2+^_o_ concentrations were then assayed by flow cytometry (9,11).

HEK-CaSR cells expressing each of the six Arg15 missense AP2σ2 variants (i.e. the three observed Arg15Cys, Arg15His and Arg15Leu and the three non-observed Arg15Gly, Arg15Pro and Arg15Ser AP2σ2 variants) were found to have half-maximal effective concentration (EC_50_) values that were significantly higher (*P* < 0.0001) than cells expressing the wild-type AP2σ2 protein. Thus, the three non-observed Arg15 AP2σ2 variants decreased the sensitivity of HEK-CaSR cells to Ca^2+^_o_ concentrations in a similar manner to the three FHH3-causing AP2σ2 mutations (Fig. 5 and Table 2). However, cells expressing the non-observed Arg15Gly, Arg15Pro or Arg15Ser AP2σ2 mutants were found to have significantly reduced increases in cell numbers over a 24-h period, when compared with cells expressing wild-type or FHH3-mutant AP2σ2 proteins (Fig. 5). These results indicate that the Arg15Gly, Arg15Pro and Arg15Ser AP2σ2 mutants are likely not observed as they are associated with an impairment of cell growth, when compared with wild-type or the FHH3-associated mutant AP2σ2 proteins (Fig. 5 and Supplementary Material, Fig. S1).

**Table 2.** EC_50_ values of observed and non-observed AP2σ2 Arg15 mutants

AP2σ2 construct	EC_50_ (mm)			
	Mean value	95% CI	*N*	*P*-value (versus WT)
pBI-CMV4 vector	2.85	2.80–2.89	10	NS
Wild-type AP2σ2	2.77	2.74–2.81	10	—
Observed AP2σ2 mutants				
Arg15His	3.02	2.96–3.08	9	<0.0001
Arg15Cys	3.03	2.98–3.07	10	<0.0001
Arg15Leu	3.07	3.01–3.13	10	<0.0001
Non-observed AP2σ2 mutants				
Arg15Gly	2.99	2.94–3.04	9	<0.0001
Arg15Pro	2.92	2.85–2.98	8	<0.0001
Arg15Ser	2.94	2.89–3.00	8	<0.0001

The number (*N*) of replicate experiments from three independent transfections is indicated.

NS, not significant; CI, confidence interval.

### Dominant-negative effects of AP2S1 mutations

**Figure 6.**
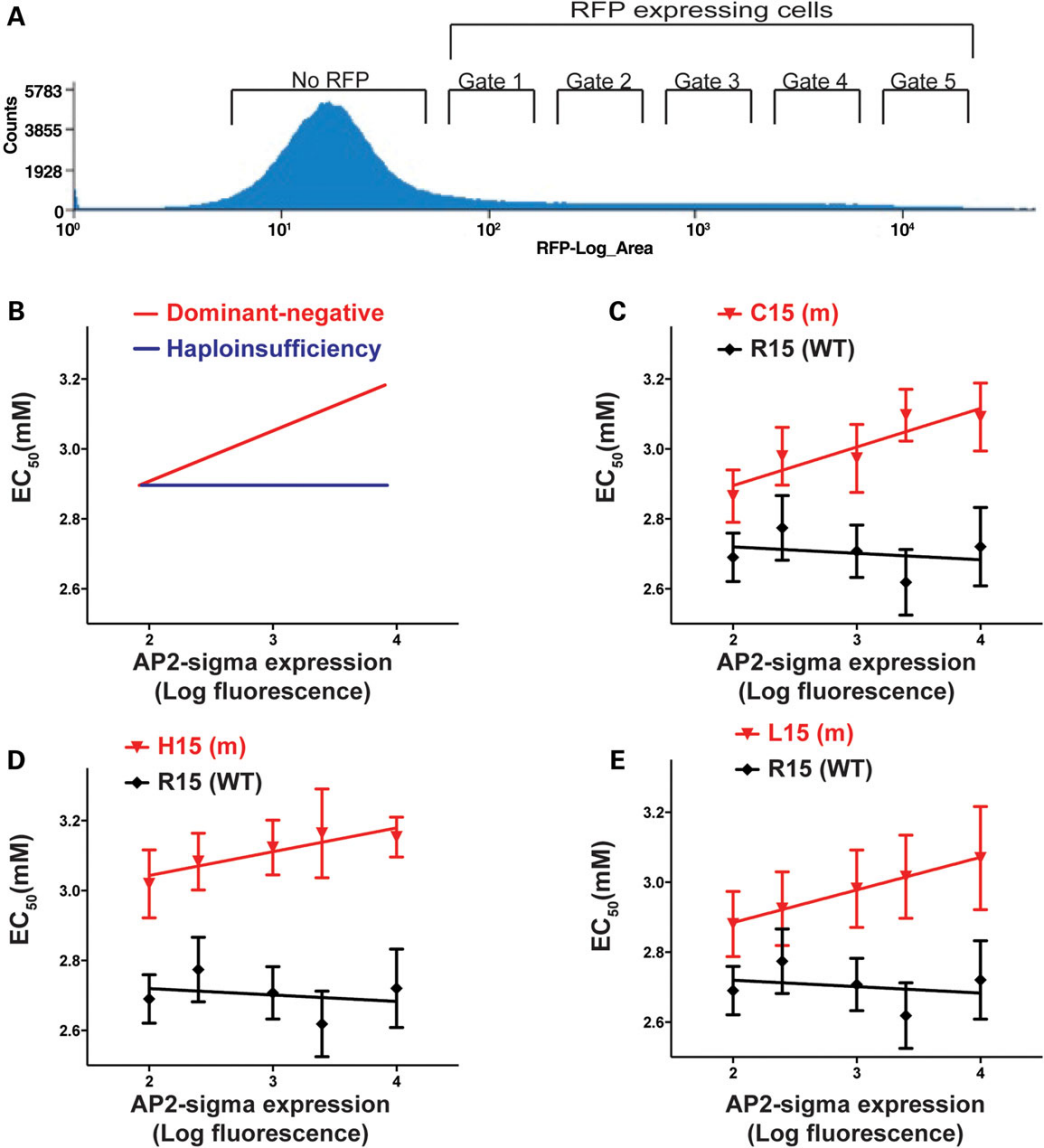
Comparison of EC_50_ values for HEK-CaSR cells transfected with increasing amounts of wild-type (WT) or mutant *AP2S1*-pBI-CMV4-RFP expression constructs. (**A**) During flow cytometry, fluorescence over a 1–10 000 range can be detected. The first large peak represents cells not expressing RFP but displaying low levels of auto-fluorescence. Cells displaying increased fluorescence with respect to this population reflect those expressing RFP. Cells from five different RFP fluorescences were selected on the basis of mean fluorescence (gate 1 log mean fluorescence = 50–100, gate 2 log mean fluorescence = 125–250, gate 3 log mean fluorescence = 500–1000, gate 4 log mean fluorescence = 1250–2500 and gate 5 log mean fluorescence = 5000–10000), consistent with a fluorescence ranging from between 1- and 100-fold over baseline, i.e. between gate 1 and gate 5, which represent the lowest and the highest levels of RFP fluorescence, and therefore AP2σ2, respectively. (**B**) Predicted linear regressions of mean EC_50_ with effects owing to dominant-negative and haploinsufficiency mutants. Loss-of-function mutants with dominant-negative effects on the WT protein will exert greater effects on EC_50_ values with increasing concentrations (red line), whereas loss-of-function mutants associated with haploinsufficiency of the WT protein will not affect the EC_50_ values with increasing concentrations as the EC_50_ will depend on the concentration of WT protein, which will remain constant (blue line). (**C–E**) Linear regression of the mean EC_50_ of cells with increasing levels of AP2σ2 expression demonstrate no significant deviation from zero in cells transfected with the WT-R15 *AP2S1*-pBI-CMV4-RFP expression construct (*r*^2^ = 0.07, *P* = 0.67), whereas a significantly positive incline to the slope is observed in cells transfected with the mutant *AP2S1-*pBI-CMV4-RFP expression constructs (C15 *r*^2^ = 0.82, *P* < 0.05; H15 *r*^2^ = 0.84, *P* < 0.05; L15 *r*^2^ = 0.99, *P* < 0.0001), thereby indicating that FHH3-associated AP2σ2 mutations may act in a dominant-negative manner. All experiments were conducted on *N* = 8 separate occasions.

FHH3 is associated with heterozygous loss-of-function AP2σ2 mutations, and these could be causing the disease by either haploinsufficiency (i.e. a reduced dosage of the wild-type AP2σ2 protein) or dominant-negative effects on the heterotetrameric AP2 complex. To investigate these genetic mechanisms, we studied the effects of altering the dosage of wild-type and mutant AP2σ2 proteins on the EC_50_ responses of HEK-CaSR cells that were transiently transfected with wild-type or mutant *AP2S1*-pBI-CMV4-RFP expression constructs. We used the bidirectional *AP2S1*-pBI-CMV4-RFP vector as it expresses RFP and AP2σ2 at equivalent levels, thereby enabling RFP expression to be used as a surrogate for AP2σ2 expression (9). We selected populations of cells with increasing levels of RFP expression by flow cytometry (Fig. 6) and assessed the effects of altering wild-type and mutant AP2σ2 dosage by measuring the Ca^2+^_i_ responses of the HEK-CaSR cells to changes in Ca^2+^_o_ concentrations, and determining the linear regression of mean EC_50_ values against expression levels of wild-type and mutant AP2σ2 proteins. In this experiment, loss-of-function mutations with dominant-negative actions, which would exert greater effects with increasing protein concentrations, will show a positive correlation between mutant protein concentration and EC_50_. However, loss-of-function mutations associated with haploinsufficiency would not exert effects on the wild-type protein, whose concentration would remain constant, and hence there would be absent correlation between mutant protein concentrations and EC_50_ values (Fig. 6).

The results of such linear regression analyses revealed that increased expression levels of FHH3-causing Arg15-mutant AP2σ2 proteins progressively impaired the sensitivity of HEK-CaSR cells, as highlighted by a significantly positive relationship between the expression levels of all three AP2σ2 mutants and the mean cellular EC_50_ responses (Cys15 *r*^2^ = 0.82, *P* < 0.05; His15 *r*^2^ = 0.84, *P* < 0.05; Leu15 *r*^2^ = 0.99, *P* < 0.0001), but that increased expression levels of wild-type AP2σ2 had no effect on EC_50_ values (*r*^2^ = 0.07, *P* = 0.67) (Fig. 6). These findings indicate a potential dominant-negative effect of the FHH3-causing Arg15 AP2σ2 mutants.

## Discussion

Our results, which have identified *AP2S1* mutations that only result in missense substitutions of the Arg15 residue in 17 additional FHH probands, have helped to establish a genotype–phenotype correlation between AP2σ2 mutants and the severity of hypercalcaemia; devise an index based on measurements of plasma calcium and magnesium concentrations and urinary clearances of calcium and creatinine to differentiate between FHH1 and FHH3; elucidate the occurrence of a mutational bias at the AP2σ2 Arg15 residue in FHH3 patients and define the likely genetic mechanism for AP2σ2 mutations as being a dominant-negative action in causing FHH3. In addition, our study shows that FHH3 can be associated with *de novo AP2S1* mutations, consistent with a previous report (12), thereby indicating that patients may not have a family history of the disorder and that *AP2S1* mutations may be associated with non-familial forms of hypercalcaemia and hypocalciuria.

The identification by this study of *AP2S1* mutations in 17 additional probands with FHH3, together with ours and other previous reports (11,12,14), yields a total of 36 probands with such AP2σ2 mutations, which all involve the Arg15 residue and result in 1 of 3 missense substitutions comprising Arg15Cys, Arg15His and Arg15Leu. Our analyses of these subjects reveal genotype–phenotype correlations for these AP2σ2 mutations, in which the Arg15Leu mutation is associated with the most pronounced hypercalcaemia, whereas Arg15His is associated with the mildest increase in serum calcium concentrations. This is surprising and the mechanisms underlying these genotype–phenotype correlations are unclear, as three-dimensional modelling and *in vitro* functional expression studies indicate that all three Arg15 AP2σ2 mutations disrupt CaSR activity to a similar extent (11), and the basis for the differences between our *in vivo* clinical observations and *in vitro* results remains to be elucidated.

**Figure 7.**
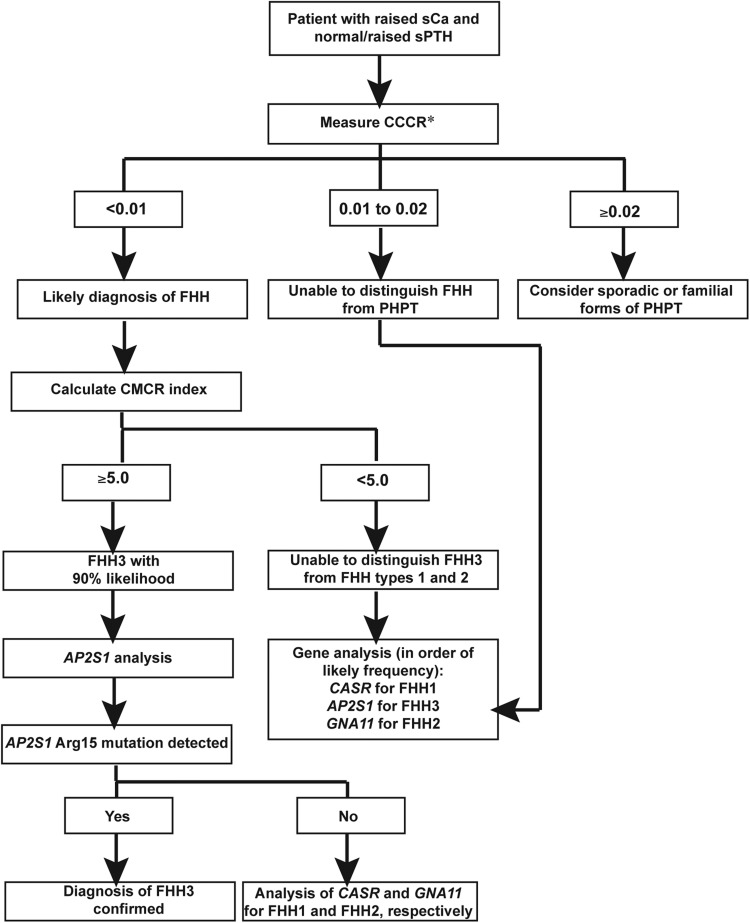
Clinical approach to distinguishing between FHH1 and FHH3 in a hypercalcaemic patient. sCa, serum calcium; sMg, serum magnesium; sPTH, serum PTH; CCCR, calcium to creatinine clearance ratio; CMCR = sCa × sMg/100 × CCCR; PHPT; primary hyperparathyroidism. *In a hypercalcaemic patient with normal/raised sPTH, a CCCR of <0.01 is consistent with a diagnosis of FHH, provided that thiazide diuretic use, vitamin D deficiency and renal impairment have been excluded.

Our study has also revealed phenotypic differences between FHH1 and FHH3. In particular, FHH3 was associated with significantly greater elevations of serum calcium, and >20% of FHH3 patients in this cohort had symptomatic hypercalcaemia. These findings are not typical of FHH, which is considered to be a benign and asymptomatic disorder, and in general associated with serum calcium concentrations that remain within 10% of the upper limit of the normal range (1). In addition to the more severe hypercalcaemia, FHH3 was associated with more pronounced suppression of urinary calcium excretion than FHH1. The more marked serum and urinary calcium phenotype of FHH3 contrasts with the results of *in vitro* studies that indicate FHH3-causing *AP2S1* mutations to lead to a milder shift in the set point of CaSR-expressing cells when compared with FHH1-causing *CASR* mutations (11). These findings suggest that AP2σ2 mutations may influence Ca^2+^_o_ homeostasis through effects on cell membrane proteins other than the CaSR. Indeed, the renal thick ascending limb Na^+^/K^+^/2Cl^−^ (NKCC2) transporter, which is pivotal for urinary calcium reabsorption, has been demonstrated to be regulated by clathrin-mediated endocytosis (24). Other phenotypic differences between FHH3 and FHH1 included the occurrence of low BMD and cognitive dysfunction in FHH3. Low BMD occurred in five FHH3 probands with *AP2S1* mutations whereas patients with FHH1, owing to *CASR* mutations, have been reported to have normal bone resorption rates and BMD measurements at the spine, hip and forearm (25). Cognitive dysfunction was observed in seven FHH3 probands, four of which presented with serum calcium concentrations of >3.0 mmol/l, and exposure to marked hypercalcaemia in infancy or childhood may have led to developmental delay and adversely affected neurological development in these individuals (26). It is also possible that expression of mutant AP2σ2 subunits within the brain may directly influence neurological development, consistent with the role of the AP2 complex in mediating receptor trafficking within neuronal synapses of the hippocampus (27), a region of the brain required for memory acquisition and spatial orientation. The Arg15Leu *AP2S1* mutation was also associated with recurrent pancreatitis in one FHH3 proband, a finding consistent with FHH1, in which recurrent pancreatitis has occasionally been reported (28). However, FHH1 probands with pancreatitis typically harbour heterozygous mutations of both the *CASR* and serine protease inhibitor, Kazal type 1 (*SPINK1)* genes (29). In contrast, the affected FHH3 proband did not harbour a mutation of *SPINK1* or other genes associated with pancreatitis (19), and this proband's marked hypercalcaemia may have been sufficient to disrupt pancreatic function. Given these possible phenotypic differences between FHH3 and FHH1, we sought to design an index based on clinical biochemistry tests of serum and urine that would help to differentiate the two disorders. The findings that FHH3 is associated with greater elevations in serum calcium and magnesium concentrations, and reductions in urinary calcium excretion, when compared with FHH1, led us to design the calcium–magnesium–calcium clearance ratio (CMCR), calculated as sCa × sMg/100 × CCCR. Assessment of the performance characteristics of this index indicated that FHH patients with CMCR ≥5.0 were significantly more likely to have FHH3. Thus, the CMCR index may have utility in the clinical setting for directing FHH patients for either *CASR* or *AP2S1* gene analysis, with patients having a CMCR value of ≥5.0 being prioritized for *AP2S1* analysis (Fig. 7). However, ∼30% of FHH3 patients have CMCR values that overlap with that of FHH1 patients (Fig. 3), and combined analysis of the *CASR*, *AP2S1* and *GNA11* genes is suggested in individuals with CMCR values of <5.0 (Fig. 7). No correlation was observed between the CMCR index and codon 15 genotype of the FHH3 patients. The CMCR index requires further validation in studies of other populations and using alternate biochemical assays.

The finding that all the 36 reported FHH3 patients, of which 17 are from this study, harbour either an Arg15Cys, Arg15His or Arg15Leu mutation, highlights the importance of this evolutionary conserved residue for AP2-mediated Ca^2+^_o_ homeostasis (11,12,14). Furthermore, the absence of the other possible missense substitutions at this codon (Arg15Gly, Arg15Pro or Arg15Ser) indicates mutation bias at the AP2σ2 Arg15 residue. Our *in vitro* characterization of the non-observed Gly15, Pro15 or Ser15 mutations demonstrated these mutant AP2σ2 proteins to be expressed and to result in an impairment of CaSR activity. However, the Gly15, Pro15 or Ser15 mutants had additional deleterious effects that led to a reduction in the numbers of cells expressing these mutants. These findings indicate a potential role for the AP2 complex in cell growth or viability and are consistent with a previously reported study of mice harbouring a germline ablation of the AP2μ2 subunit, which led to early embryonic lethality, thus highlighting the involvement of the ubiquitously expressed AP2 complex in mammalian development (30). Thus, a possible explanation for the absence of the Arg15Gly, Arg15Pro and Arg15Ser AP2σ2 mutations in patients is that these deleterious mutations likely result in embryonic lethality, whereas the FHH3-causing AP2σ2 mutations Arg15Cys, Arg15His and Arg15Leu are observed in patients, as these mutations are not associated with deleterious effects on cell growth, but are tolerated and compatible with embryonic and post-natal survival.

Our findings of a likely dominant-negative effect of FHH3-causing Arg15 AP2σ2 mutations are consistent with previous mutagenesis studies of the AP2 complex (31). Indeed, overexpression of a mutated μ2-subunit (AP2μ2), which displayed diminished capacity to bind to some cargo proteins, has been reported to inhibit receptor trafficking in a dominant-negative manner (31). The precise mechanisms of such dominant-negative actions remain to be established but three possibilities are (1) the incorporation of a mutant σ2 subunit may impair the assembly of the tetrameric complex, (2) incorporation of the mutant σ2 subunit may alter the structure of the AP2 complex and hinder the efficiency of wild-type AP2α, AP2β and AP2μ subunits (32) and (3) the mutant AP2 complex may potentially sequester the CaSR at the plasma membrane and prevent wild-type AP2 molecules from effectively trafficking this GPCR.

In summary, our studies of *AP2S1* mutations, which highlight FHH3 as a distinct disorder of Ca^2+^_o_ homeostasis and demonstrate the importance of the AP2σ2 Arg15 residue for AP2 function, have revealed genotype–phenotype correlations with mutation bias at the Arg15 residue, as well as a likely dominant-negative mechanism of action.

## Materials and Methods

### Subjects

Informed consent was obtained from individuals, using protocols approved by the local and national ethics committees (MREC/02/2/93). Sixty-five unrelated probands (22 males and 43 females) were ascertained. The age at diagnosis or presentation ranged from early infancy to 76 years, and all probands had hypercalcaemia with normal or elevated PTH concentrations, in association with inappropriately low urinary calcium excretion. Previous mutational analysis of *CASR* and *GNA11* had not identified any abnormalities of the coding regions and exon–intron boundaries of these genes (8,9).

### DNA sequence analysis

Leukocyte DNA was extracted from venous blood samples and quantified using the High sensitivity Qubit system (Invitrogen) (11). *AP2S1*-specific primers were used to perform PCR amplification of the five exons and eight intron-exon boundaries of the *AP2S1* gene, as previously reported (11). DNA sequence analysis of the PCR products was performed using the BigDye Terminator v3.1 Cycle Sequencing Kit (Life Technologies) and an ABI automated capillary sequencer (Applied Biosystems), as reported (8). DNA sequence abnormalities and co-segregation in families were confirmed by restriction endonuclease analysis (New England Biolabs), as described (11).

### Computer modelling of the AP2 structure

The crystal structure of the AP2 heterotetramer bound to an acidic dileucine peptide has been reported previously (18). The PyMOL Molecular Graphics System (version 1.2r3pre, Schrödinger) was used to model the effect of the FHH3-associated AP2σ2 mutations on the interaction with the acidic dileucine motif peptide using the three-dimensional structure of AP2 archived in the Protein Data Bank at the European Bioinformatics Institute with the accession number 2JKR (8,9,11,18). The effect of the Arg15Cys, Arg15Leu, Arg15His, Arg15Pro, Arg15Ser and Arg15Gly mutations on the interaction of AP2σ2 subunit with the acidic dileucine peptide was modelled using PyMod plug-in and Modeller.21 (9).

### Generation of *AP2S1* expression constructs

An AP2σ2 expression construct was generated by cloning the full-length *AP2S1* coding region into the bidirectional cloning vector pBI-CMV4-RFP (Clontech), which allows for co-expression of AP2σ2 and RFP at equivalent levels (11,33). The use of RFP minimized overlap between the emission spectra of this reporter gene and the indo-1-AM Ca^2+^-binding fluorophore. *AP2S1* mutations were introduced into the construct by site-directed mutagenesis (QuikChange Lightning, Stratagene) and confirmed by DNA sequence analysis, as described (8,11).

### Cell culture and transfection

Functional studies of *AP2S1* mutations were performed in HEK293 cells that stably expressed the CaSR (11). HEK293 cells were used because suitable parathyroid and renal tubular cells are not available, and HEK293 cells have been established as a model for such studies (8,9,11,34). The reported stably CaSR-transfected HEK293 cell line (HEK-CaSR) (9,11) was cultured in high-glucose DMEM (Invitrogen) supplemented with 10% fetal bovine serum and 1% geneticin (11). A high level of CaSR expression in these cells was confirmed by western blot analysis of whole-cell protein extract using a mouse monoclonal antibody to human CaSR (ADD; Abcam, ab19347, 1:1000) (9,11). The wild-type and mutant AP2σ2 constructs were transiently transfected into HEK-CaSR cells using Lipofectamine 2000 (Invitrogen) (9,11). Expression of RFP was used as a surrogate for AP2σ2 expression as it is expressed at equivalent levels to AP2σ2 by the pBI-CMV4-RFP bidirectional vector (11). Western blot analysis of cellular protein extract was undertaken using a rabbit polyclonal antibody to RFP (Thermo Scientific, PA1-986, 1:500) (11). The membrane was re-probed with mouse anti-GAPDH antibody (Abcam, ab8245, 1:3000) as a loading control. Successful transfection was also confirmed by visualising RFP fluorescence using an Eclipse E400 fluorescence microscope with an epifluorescence filter, and images were captured using a DXM1200C digital camera and NIS Elements software (Nikon) (9,11). The effect of mutant AP2σ2 proteins on cell growth was assessed by determining the percentage increase in cell numbers over a 24-h period, as follows. Twenty-four hours after transfection, equal numbers of cells were seeded into a 96-well plate, and the cells were imaged, at 48 and 72 h, using fluorescence microscopy, as mentioned above (9,11). Following blinding to both transfection and time point, the numbers of transfected cells were counted manually at 48 and 72 h post-transfection, and the percentage increase in cells determined using Microsoft Excel and statistical analysis undertaken in GraphPad Prism (GraphPad) (9,11).

### Measurement of Ca^2+^_i_ responses

The effect of mutant AP2σ2 proteins on CaSR-mediated Ca^2+^_i_ responses were assessed by determining EC_50_ values (i.e. [Ca^2+^]_o_ required for 50% of the maximal response) and comparing these to the wild-type EC_50_, as reported (8,9,11). Briefly, 48 h after transfection, the cells were harvested, washed in calcium- and magnesium-free Hank's balanced salt solution (HBSS) (Invitrogen) and loaded with 1 μg/ml indo-1-acetoxymethylester (Indo-1-am) (Molecular Probes) for 1 h at 37 °C (8,9,11). After the removal of free dye, the cells were resuspended in calcium- and magnesium-free HBSS and maintained at 37°C. Flow cytometry was performed with a Beckman Coulter MoFlo XDP equipped with JDSUY Xcyte UV Laser and a Coherent Sapphire 488 Laser using a 550LP dichroic mirror and 580/30 bandpass filter. Single cells were isolated from debris on the basis of morphology using forward scatter and side scatter readings (8,9,11). Cells were stimulated by sequentially adding calcium to the calcium- and magnesium-free HBSS to progressively increase the [Ca^2+^]_o_ from 0 to 15 mm. The baseline fluorescence ratio was measured for 2 min, the fluorescence ratio compared with the time was recorded and data were collected for 2 min at each [Ca^2+^]_o_. Cytomation Summit software was used to determine the peak mean fluorescence ratio of the transient response after each individual stimulus expressed as a normalized response (8,9,11). Analysis of five separate populations of cells gated for increasing RFP as a concordant surrogate for increasing AP2σ2 expression was undertaken and concentration–response curves generated using the normalized response at each of nine different [Ca^2+^]_o_ (0–15 mm) for each separate experiment. Nonlinear regression of the concentration–response curves was performed with GraphPad Prism (GraphPad) to calculate the EC_50_ for each separate experiment (9,11). Subsequent linear regression of the mean EC_50_ (*N* = 8 experiments) for each *AP2S1*-pBI-CMV4-RFP expression vector was undertaken with GraphPad Prism (GraphPad) (9,11).

### Statistical analyses

The phenotypic data from this study were pooled with those of our previous studies of FHH3 patients and families (11,20,23). To undertake a comparison of phenotypes between FHH3 and FHH1, we used data from 43 previously reported unrelated FHH1 probands (13 males and 30 females, aged 1–84 years) who had germline heterozygous *CASR* mutations (8). Comparisons of continuous variables between two groups were undertaken using the Mann–Whitney *U* test, and the Kruskal–Wallis test was used to compare multiple groups. Categorical variables were analysed using the Chi-squared test. ROC analysis using values of the CMCR sensitivity and specificity was performed to study the discriminatory power of the CMCR index to distinguish between FHH1 and FHH3. Comparisons of EC_50_ values were performed using the *F* test, as described (9,11). The Mann–Whitney *U* test was used to compare the proliferation rates of cells expressing wild-type or mutant AP2σ2 proteins. All analyses were undertaken using GraphPad Prism (GraphPad) and are presented as mean ± SEM unless otherwise stated. A value of *P* < 0.05 was considered significant for all analyses.

## Supplementary Material

Supplementary Material is available at *HMG* online.

## Funding

This work was supported by the United Kingdom Medical Research Council (MRC) programme grants—G9825289 and G1000467 (to M.A.N., F.M.H. and R.V.T.) and National Institute for Health Research (NIHR) Oxford Biomedical Research Centre Programme (to M.A.N. and R.V.T.). S.A.H. and A.R. are Wellcome Trust Clinical Training Fellows. Funding to pay the Open Access publication charges for this article was provided by Medical Research Council (MRC), UK and Wellcome Trust Open Access Block Grants.

## Supplementary Material

Supplementary Data

## References

[1] Hannan F.M., Thakker R.V. (2013) Calcium-sensing receptor (CaSR) mutations and disorders of calcium, electrolyte and water metabolism. Best. Pract. Res. Clin. Endocrinol. Metab., 27, 359–371. 23856265 10.1016/j.beem.2013.04.007

[2] Marx S.J., Spiegel A.M., Brown E.M., Koehler J.O., Gardner D.G., Brennan M.F., Aurbach G.D. (1978) Divalent cation metabolism. Familial hypocalciuric hypercalcemia versus typical primary hyperparathyroidism. Am. J. Med., 65, 235–242. 686009 10.1016/0002-9343(78)90814-8

[3] Christensen S.E., Nissen P.H., Vestergaard P., Heickendorff L., Brixen K., Mosekilde L. (2008) Discriminative power of three indices of renal calcium excretion for the distinction between familial hypocalciuric hypercalcaemia and primary hyperparathyroidism: a follow-up study on methods. Clin. Endocrinol. (Oxf), 69, 713–720. 18410554 10.1111/j.1365-2265.2008.03259.x

[4] Christensen S.E., Nissen P.H., Vestergaard P., Heickendorff L., Rejnmark L., Brixen K., Mosekilde L. (2008) Plasma 25-hydroxyvitamin D, 1,25-dihydroxyvitamin D, and parathyroid hormone in familial hypocalciuric hypercalcemia and primary hyperparathyroidism. Eur. J. Endocrinol., 159, 719–727. 18787045 10.1530/EJE-08-0440

[5] Pollak M.R., Brown E.M., Chou Y.H., Hebert S.C., Marx S.J., Steinmann B., Levi T., Seidman C.E., Seidman J.G. (1993) Mutations in the human Ca(2+)-sensing receptor gene cause familial hypocalciuric hypercalcemia and neonatal severe hyperparathyroidism. Cell, 75, 1297–1303. 7916660 10.1016/0092-8674(93)90617-y

[6] Pearce S.H., Trump D., Wooding C., Besser G.M., Chew S.L., Grant D.B., Heath D.A., Hughes I.A., Paterson C.R., Whyte M.P. et al (1995) Calcium-sensing receptor mutations in familial benign hypercalcemia and neonatal hyperparathyroidism. J. Clin. Invest., 96, 2683–2692. 8675635 10.1172/JCI118335 PMC185975

[7] Pearce S.H., Bai M., Quinn S.J., Kifor O., Brown E.M., Thakker R.V. (1996) Functional characterization of calcium-sensing receptor mutations expressed in human embryonic kidney cells. J. Clin. Invest., 98, 1860–1866. 8878438 10.1172/JCI118987 PMC507626

[8] Hannan F.M., Nesbit M.A., Zhang C., Cranston T., Curley A.J., Harding B., Fratter C., Rust N., Christie P.T., Turner J.J. et al (2012) Identification of 70 calcium-sensing receptor mutations in hyper- and hypo-calcaemic patients: evidence for clustering of extracellular domain mutations at calcium-binding sites. Hum. Mol. Genet., 21, 2768–2778. 22422767 10.1093/hmg/dds105

[9] Nesbit M.A., Hannan F.M., Howles S.A., Babinsky V.N., Head R.A., Cranston T., Rust N., Hobbs M.R., Heath H. 3rd, Thakker R.V. (2013) Mutations affecting G-protein subunit alpha11 in hypercalcemia and hypocalcemia. N. Engl. J. Med., 368, 2476–2486. 23802516 10.1056/NEJMoa1300253 PMC3773604

[10] Hofer A.M., Brown E.M. (2003) Extracellular calcium sensing and signalling. Nat. Rev. Mol. Cell. Biol., 4, 530–538. 12838336 10.1038/nrm1154

[11] Nesbit M.A., Hannan F.M., Howles S.A., Reed A.A., Cranston T., Thakker C.E., Gregory L., Rimmer A.J., Rust N., Graham U. et al (2013) Mutations in AP2S1 cause familial hypocalciuric hypercalcemia type 3. Nat. Genet., 45, 93–97. 23222959 10.1038/ng.2492 PMC3605788

[12] Fujisawa Y., Yamaguchi R., Satake E., Ohtaka K., Nakanishi T., Ozono K., Ogata T. (2013) Identification of AP2S1 mutation and effects of low calcium formula in an infant with hypercalcemia and hypercalciuria. J. Clin. Endocrinol. Metab., 98, E2022–E2027. 24081735 10.1210/jc.2013-2571

[13] Hendy G.N., Cole D.E. (2013) Ruling in a suspect: the role of AP2S1 mutations in familial hypocalciuric hypercalcemia type 3. J. Clin. Endocrinol. Metab., 98, 4666–4669. 24311792 10.1210/jc.2013-3616 PMC3849666

[14] Hendy G.N., Canaff L., Newfield R.S., Tripto-Shkolnik L., Wong B.Y., Lee B.S., Cole D.E. (2014) Codon Arg15 mutations of the AP2S1 gene: common occurrence in familial hypocalciuric hypercalcemia cases negative for calcium-sensing receptor (CASR) mutations. J. Clin. Endocrinol. Metab., 99, E1311–E1315. 24731014 10.1210/jc.2014-1120

[15] Collins B.M., McCoy A.J., Kent H.M., Evans P.R., Owen D.J. (2002) Molecular architecture and functional model of the endocytic AP2 complex. Cell, 109, 523–535. 12086608 10.1016/s0092-8674(02)00735-3

[16] Edeling M.A., Mishra S.K., Keyel P.A., Steinhauser A.L., Collins B.M., Roth R., Heuser J.E., Owen D.J., Traub L.M. (2006) Molecular switches involving the AP-2 beta2 appendage regulate endocytic cargo selection and clathrin coat assembly. Dev. Cell., 10, 329–342. 16516836 10.1016/j.devcel.2006.01.016

[17] Ohno H. (2006) Physiological roles of clathrin adaptor AP complexes: lessons from mutant animals. J. Biochem., 139, 943–948. 16788044 10.1093/jb/mvj120

[18] Kelly B.T., McCoy A.J., Spate K., Miller S.E., Evans P.R., Honing S., Owen D.J. (2008) A structural explanation for the binding of endocytic dileucine motifs by the AP2 complex. Nature, 456, 976–979. 19140243 10.1038/nature07422 PMC4340503

[19] Whitcomb D.C. (2010) Genetic aspects of pancreatitis. Ann. Rev. Med., 61, 413–424. 20059346 10.1146/annurev.med.041608.121416

[20] McMurtry C.T., Schranck F.W., Walkenhorst D.A., Murphy W.A., Kocher D.B., Teitelbaum S.L., Rupich R.C., Whyte M.P. (1992) Significant developmental elevation in serum parathyroid hormone levels in a large kindred with familial benign (hypocalciuric) hypercalcemia. Am. J. Med., 93, 247–258. 1524075 10.1016/0002-9343(92)90229-5

[21] Lloyd S.E., Pannett A.A., Dixon P.H., Whyte M.P., Thakker R.V. (1999) Localization of familial benign hypercalcemia, Oklahoma variant (FBHOk), to chromosome 19q13. Am. J. Hum. Genet., 64, 189–195. 9915958 10.1086/302202 PMC1377717

[22] Hannan F.M., Nesbit M.A., Turner J.J., Stacey J.M., Cianferotti L., Christie P.T., Conigrave A.D., Whyte M.P., Thakker R.V. (2010) Comparison of human chromosome 19q13 and syntenic region on mouse chromosome 7 reveals absence, in man, of 11.6 Mb containing four mouse calcium-sensing receptor-related sequences: relevance to familial benign hypocalciuric hypercalcaemia type 3. Eur. J. Hum. Genet., 18, 442–447. 19809483 10.1038/ejhg.2009.161 PMC2842244

[23] Nesbit M.A., Hannan F.M., Graham U., Whyte M.P., Morrison P.J., Hunter S.J., Thakker R.V. (2010) Identification of a second kindred with familial hypocalciuric hypercalcemia type 3 (FHH3) narrows localization to a <3.5 megabase pair region on chromosome 19q13.3. J. Clin. Endocrinol. Metab., 95, 1947–1954. 20133464 10.1210/jc.2009-2152

[24] Ares G.R., Ortiz P.A. (2012) Dynamin2, clathrin, and lipid rafts mediate endocytosis of the apical Na/K/2Cl cotransporter NKCC2 in thick ascending limbs. J. Biol. Chem., 287, 37824–37834. 22977238 10.1074/jbc.M112.386425 PMC3488056

[25] Christensen S.E., Nissen P.H., Vestergaard P., Heickendorff L., Rejnmark L., Brixen K., Mosekilde L. (2009) Skeletal consequences of familial hypocalciuric hypercalcaemia versus primary Hyperparathyroidism. Clin. Endocrinol. (Oxf), 71, 798–807. 19250271 10.1111/j.1365-2265.2009.03557.x

[26] Cole D., Forsythe C.R., Dooley J.M., Grantmyre E.B., Salisbury S.R. (1990) Primary neonatal hyperparathyroidism: a devastating neurodevelopmental disorder if left untreated. J. Craniofac. Genet. Dev. Biol., 10, 205–214. 2211966

[27] Lee S.H., Liu L., Wang Y.T., Sheng M. (2002) Clathrin adaptor AP2 and NSF interact with overlapping sites of GluR2 and play distinct roles in AMPA receptor trafficking and hippocampal LTD. Neuron, 36, 661–674. 12441055 10.1016/s0896-6273(02)01024-3

[28] Pearce S.H., Wooding C., Davies M., Tollefsen S.E., Whyte M.P., Thakker R.V. (1996) Calcium-sensing receptor mutations in familial hypocalciuric hypercalcaemia with recurrent pancreatitis. Clin. Endocrinol. (Oxf), 45, 675–680. 9039332 10.1046/j.1365-2265.1996.750891.x

[29] Felderbauer P., Klein W., Bulut K., Ansorge N., Dekomien G., Werner I., Epplen J.T., Schmitz F., Schmidt W.E. (2006) Mutations in the calcium-sensing receptor: a new genetic risk factor for chronic pancreatitis? Scand. J. Gastroenterol., 41, 343–348. 16497624 10.1080/00365520510024214

[30] Mitsunari T., Nakatsu F., Shioda N., Love P.E., Grinberg A., Bonifacino J.S., Ohno H. (2005) Clathrin adaptor AP-2 is essential for early embryonal development. Mol. Cell. Biol., 25, 9318–9323. 16227583 10.1128/MCB.25.21.9318-9323.2005 PMC1265839

[31] Nesterov A., Carter R.E., Sorkina T., Gill G.N., Sorkin A. (1999) Inhibition of the receptor-binding function of clathrin adaptor protein AP-2 by dominant-negative mutant mu2 subunit and its effects on endocytosis. EMBO, 18, 2489–2499. 10.1093/emboj/18.9.2489 PMC1171331 10228163

[32] Veitia R.A. (2009) Dominant negative factors in health and disease. J. Pathol., 218, 409–418. 19544283 10.1002/path.2583

[33] Fang Y., Huang C.C., Kain S.R., Li X. (1999) Use of coexpressed enhanced green fluorescent protein as a marker for identifying transfected cells. Method Enzymol., 302, 207–212. 10.1016/s0076-6879(99)02020-0 12876773

[34] Leach K., Wen A., Davey A.E., Sexton P.M., Conigrave A.D., Christopoulos A. (2012) Identification of molecular phenotypes and biased signaling induced by naturally occurring mutations of the human calcium-sensing receptor. Endocrinology, 153, 4304–4316. 22798347 10.1210/en.2012-1449

