# Supplementary material for: Adaptor protein-2 sigma subunit mutations causing familial hypocalciuric hypercalcaemia type 3 (FHH3) demonstrate genotype–phenotype correlations, codon bias and dominant-negative effects
Source: Hum Mol Genet. 2015 Jun 16;24(18):5079–92. doi: 10.1093/hmg/ddv226 (PMC4550820; doi:10.1093/hmg/ddv226)
Supplement: Supplementary Data [file supp_24_18_5079__index.html]

Adaptor protein-2 sigma subunit mutations causing familial hypocalciuric hypercalcaemia type 3 (FHH3) demonstrate genotype-phenotype correlations, codon bias and dominant-negative effects — Adaptor protein-2 sigma subunit mutations causing familial hypocalciuric hypercalcaemia type 3 (FHH3) demonstrate genotype–phenotype correlations, codon bias and dominant-negative effects — Adaptor protein-2 sigma subunit mutations causing familial hypocalciuric hypercalcaemia type 3 (FHH3) demonstrate genotype–phenotype correlations, codon bias and dominant-negative effects — Supplementary Data 

# Adaptor protein-2 sigma subunit mutations causing familial hypocalciuric hypercalcaemia type 3 (FHH3) demonstrate genotype–phenotype correlations, codon bias and dominant-negative effects

## Supplementary Data

Supplementary Data

- Supplementary Data - Pdf file
