# Supplementary material for: Adaptor protein-2 sigma subunit mutations causing familial hypocalciuric hypercalcaemia type 3 (FHH3) demonstrate genotype–phenotype correlations, codon bias and dominant-negative effects
Source: Hum Mol Genet. 2015 Jun 16;24(18):5079–92. doi: 10.1093/hmg/ddv226 (PMC4550820; doi:10.1093/hmg/ddv226)
Supplement: Supplementary Data [file supp_ddv226_ddv226supp.pdf]

Figure S1

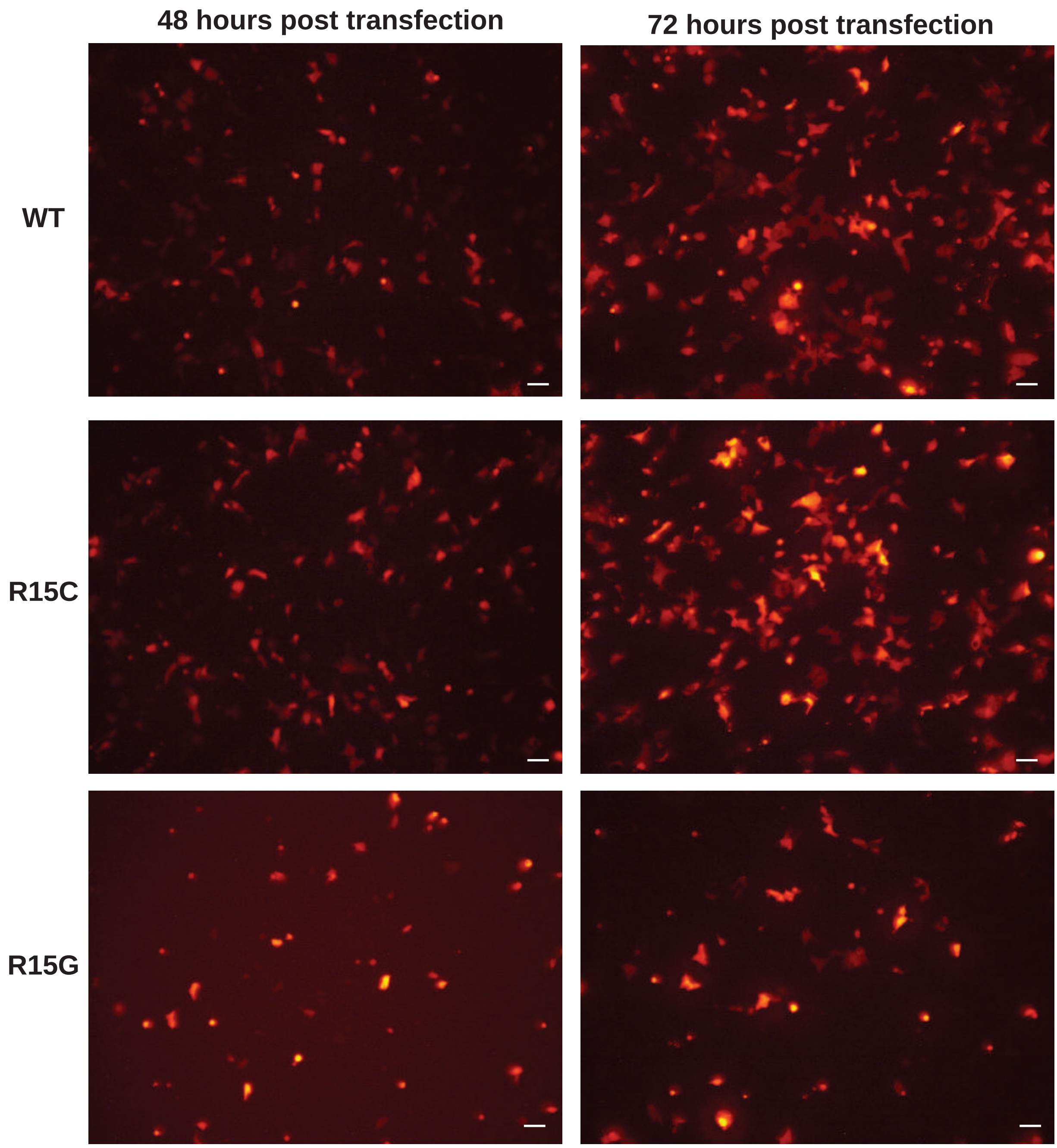

Figure S1. Representative images taken from cell proliferation assay demonstrating an equivalent increase in the number of RFP expressing cells, between 48 and 72 hours post transfection in both WT-AP2S1 and R15C-AP2S1 transfections. In contrast there is a lesser increase in the number of RFP expressing cells in the case of R15G-AP2S1 transfection. Bar indicates 20μm.
